# Hik36–Hik43 and Rre6 act as a two-component regulatory system to control cell aggregation in *Synechocystis* sp. PCC6803

**DOI:** 10.1038/s41598-020-76264-2

**Published:** 2020-11-10

**Authors:** Kota Kera, Yuichiro Yoshizawa, Takehiro Shigehara, Tatsuya Nagayama, Masaru Tsujii, Saeko Tochigi, Nobuyuki Uozumi

**Affiliations:** Department of Biomolecular Engineering, Graduate School of Engineering, Tohoku University, Aobayama 6-6-07, Sendai, 980-8579 Japan

**Keywords:** Biological techniques, Cell biology, Microbiology

## Abstract

In response to environmental stress the model cyanobacterium, *Synechocystis* sp. PCC6803 can switch from a planktonic state to autoaggregation and biofilm formation. The precise mechanism of this transition remains unknown. Here we investigated the role of a candidate two-component regulatory system (TCS) in controlling morphological changes, as a way to understand the intermediate molecular steps that are part of the signaling pathway. A bacterial two-hybrid assay showed that the response regulator Rre6 formed a TCS together with a split histidine kinase consisting of Hik36 and Hik43. Individual disruption mutants displayed autoaggregation in a static culture. In contrast, unlike in the wild type, high salinity did not induce biofilm formation in *Δhik36*, *Δhik43* and *Δrre6*. The expression levels of exopolysaccharide (EPS) production genes were higher in *Δhik36* and *Δhik43*, compared with the wild type, but lower in *Δrre6*, suggesting that the TCS regulated EPS production in *Synechocystis*. Rre6 interacted physically with the motor protein PilT2, that is a component of the type IV pilus system. This interaction was enhanced in a phosphomimic version of Rre6. Taken together, Hik36–Hik43–Rre6 function as an upstream component of the pili-related signal transduction cascade and control the prevention of cell adhesion and biofilm formation.

## Introduction

In liquid medium, bacteria generally grow as planktonic single cells. However, when they encounter environmental stress, bacteria can undergo multicellular autoaggregation. This will cause the cells to sediment and settle on the wall or bottom of the culture vessel^1^. Extracellular cell-surface structures like pili^2^, EPS, and extracellular DNA^3^ contribute to the adherence of the cells to solid materials and to the recruitment of other cells, or to the formation of aggregates in solution, resulting in clumps of cells on solid surfaces^1,4^. A biofilm is a multicellular bacterial community embedded in a self-produced extracellular polymeric matrix, which protects the cells from biotic and abiotic stress^5,6^. Bacterial cells can switch between a planktonic state and a biofilm as they adapt to various environmental cues.

In *Escherichia coli*, the life cycle is controlled by the chemosensory system that regulates flagellar chemotaxis^7^. This complex system consists of numerous components involved in chemical perception, signaling pathways and the rotation of flagella. The model cyanobacterium, *Synechocystis* sp. PCC6803 (hereafter *Synechocystis*) does not possess flagella. Instead, *Synechocystis* possesses pili that mediate cell motility required for adherence, biofilm formation and locomotion^8^. This system resembles the flagellar signal transduction pathway and has been extensively studied in *Pseudomonas aeruginosa*^9^. Light is another factor regulating autoaggregation in cyanobacteria. *Thermosynechococcus vulcanus* shows light color-dependent cell aggregation as a strategy for light avoidance^10,11^. Substrains of *Synechocystis* that display positive phototaxis (PCC-P) and negative phototaxis (PCC-N) use their type IV pilus (TFP) structure for twitching motility^12–15^.

When *Synechocystis* experiences osmotic upshock or high salinity stress, the initial response consists of controlling intracellular ion homeostasis by way of ion uptake systems, followed by the synthesis of osmolytes^16–18^. However, prolonged salinity stress leads to EPS production and biofilm formation which increases stress tolerance^19,20^. The details of the molecular mechanisms of this response are not yet understood.

Two-component regulatory systems (TCSs) are common in microbes where they function as stimulus–response mechanisms that sense changes in the environment^21,22^. TCSs consist of a sensor histidine kinase (Hik) and a response regulator (Rre). A conserved histidine residue in the histidine phosphotransfer (HPt) domain in Hik is autophosphorylated by the catalytic and ATP binding (CA) domain. The phosphoryl group is then transferred to an aspartate residue in the receiver (REC) domain of Rre. This triggers a change in the enzymatic activity, in the affinity of DNA binding and so on. In bacteria, the TCS consisting of the histidine kinase CheA and the response regulator CheY controls bacterial chemotaxis, where phosphorylated CheY interacts with the flagellar motor switch complex^23,24^. In the genome of *Synechocystis*, forty-four genes are annotated as *hik* and forty-two genes are annotated as *rre*^25,26^. *Synechocystis* has three classes of CheA-CheY-like chemotaxis-related gene clusters, the *tax1*/*pixG* cluster, the *tax2*/*sll1291* cluster and the *tax3*/*pilG* cluster^2,13,24,27,28^. The *tax1/pixG* cluster is involved in phototaxis and the *tax3*/*pilG* cluster is crucial for motility, pilus biogenesis and genetic transformation competency^13,27–29^. The *tax3*/*pilG* cluster contains *slr1041* (*taxP3*, *pilG*, *rre6*) and *slr1042* (*taxY3*, *pilH*, *rre7*)^2,13,24,27,28^. Interestingly, in the *tax3*/*pilG* cluster the *cheA* homolog is split into two genes, *slr0073* (*pilL-N*, *hik36*) and *slr0322* (*taxAY3*, *pilL-C*, *hik43*). Based on the fact that mutants in the *tax3*/*pilG* cluster showed phenotypes related to phototactic cell movement and on the overall similarity in gene arrangement pattern to CheA-CheY, it had been proposed that Hik36 and Hik43 form a single functional Hik that together with Rre7 functions as a TCS^28^.

We have previously shown that addition of high amounts of salt to *Synechocystis* cultures results in decreased polyamine content, which leads to biofilm formation^20^. To further dissect the signal transduction pathway leading to biofilm formation, we investigated whether the TCS genes in the *tax3*/*pilG* cluster mediate autoaggregation and biofilm formation in *Synechocystis*. Our analysis of protein–protein interactions and of the phenotype of TCS mutants indicated that TCS play an important role in the prevention of autoaggregation under normal condition and in the promotion of cell adhesion and biofilm formation under salt stress.

## Results

### Hik36 and Hik43 interact with Rre6 but not with Rre7

**Figure 1:**
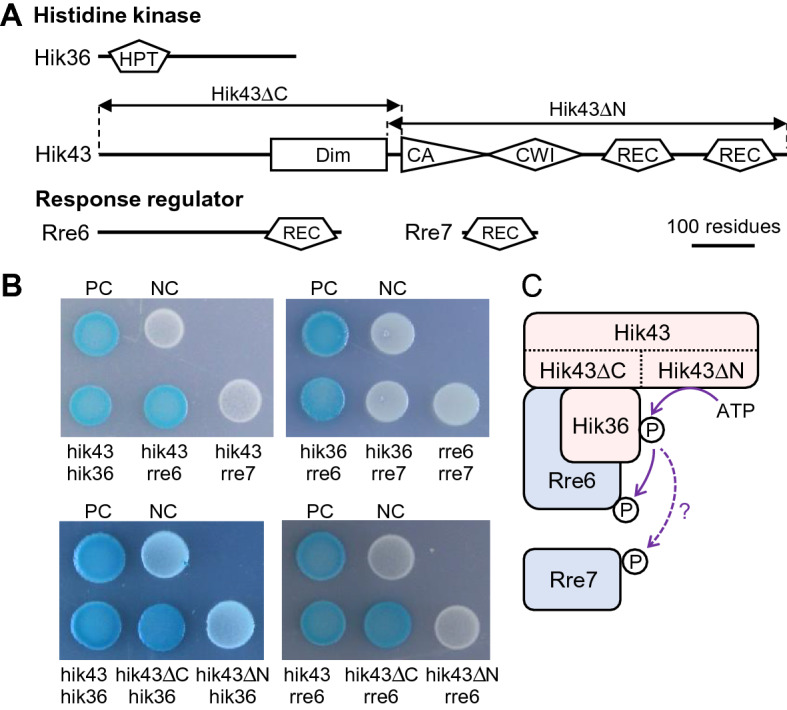
Protein–protein interactions between Hik36, Hik43 and Rre6. (**A**) Diagram showing conserved regions of histidine kinases, Hik36 and Hik43, and response regulators, Rre6 and Rre7. Motifs were predicted according to SMART (https://smart.embl-heidelberg.de). The scale bar represents 100 amino acids. HPT, histidine phosphotransfer domain; DIM, homo-dimeric domain; CA, catalytic and ATP binding domain; CWI, CheW interaction domain; REC, receiver domain. (**B**) Bacterial two-hybrid assay. Colonies were grown on solid medium containing 0.5 mM IPTG and 80 ng/mL X-Gal for 48 h. A blue colony indicates a positive interaction between the proteins. *Escherichia coli* containing pKT25-zip and pUT18C-zip were used as positive control (PC), and cells containing pKNT25 and pUT18C were used as negative control (NC). (**C**) Diagram summarizing the protein–protein interactions determined in (**B**).

The *Synechocystis tax3*/*pilG* cluster contains two *hik* genes, *hik36 (pilL-N)* and *hik43 (taxAY3*, *pilL-C)*, and two *rre* genes *rre6 (taxP3*, *pilG)* and *rre7(taxY3, pilH)*. While *hik36* and *hik43* are at distant positions in the genome, *rre6* and *rre7* are located next to each other^27,28^ (Fig. 1A and Supplemental Fig. S1). To identify protein–protein interactions between these potential components of TCS, i.e. between the histidine kinases and the response regulators, we performed a bacterial two-hybrid (BACTH) assay with Hik36, Hik43, Rre6 and Rre7^30^. *Escherichia coli* expressing combinations of Hik36–Hik43, Hik43–Rre6, and Hik36–Rre6 resulted in blue colonies, indicating direct interactions of the proteins. In contrast, no interactions were detected between Hik36–Rre7, Hik43–Rre7 and Rre6–Rre7 (Fig. 1B). These results indicated that Rre6 formed a TCS with Hik36 and Hik43 (Fig. 1C). To identify which domains of Hik43 were responsible for the interaction with Hik36 and Rre6, we generated two Hik43 variants. Hik43ΔC, lacking the CA domain, the CheW interaction domain (CWI), and the receiver domain (REC), and Hik43ΔN, lacking the homodimeric domain (DIM) (Fig. 1A). Hik43ΔC was able to interact with both Hik36 and Rre6. However, deletion of the DIM domain of Hik43 (Hik43ΔN) resulted in white colonies, indicating that the N-terminal domain was required for interaction with Hik36 and Rre6.

### Deletion of *hik36*, *hik43* or *rre6* enhanced autoaggregation and reduced salt stress-induced biofilm formation

**Figure 2:**
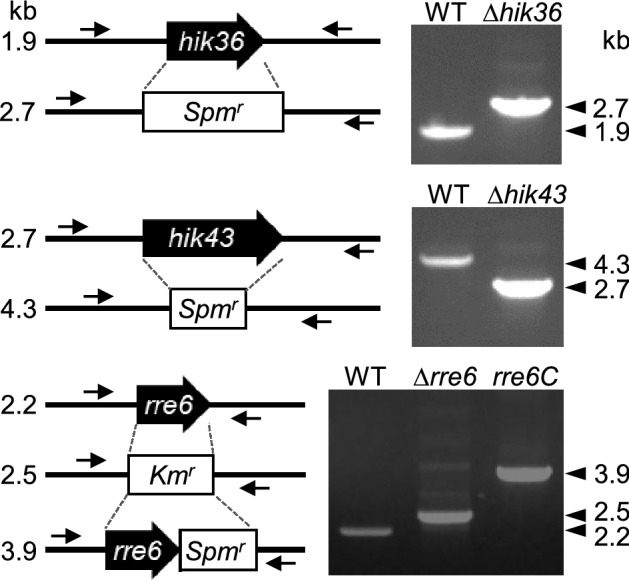
Generation of *Synechocystis* mutants. Schematic representation of disruption or reintroduction of *hik36*, *hik43* and *rre6* (left) and corresponding PCR products on an agarose gel (right). The antibiotic resistance cassettes (*Spm*^r^: Spectinomycin, *Km*^r^: Kanamycin) are shown as boxes. The size of expected PCR products is given on the left (in kb). Primers are represented by arrows.

**Figure 3:**
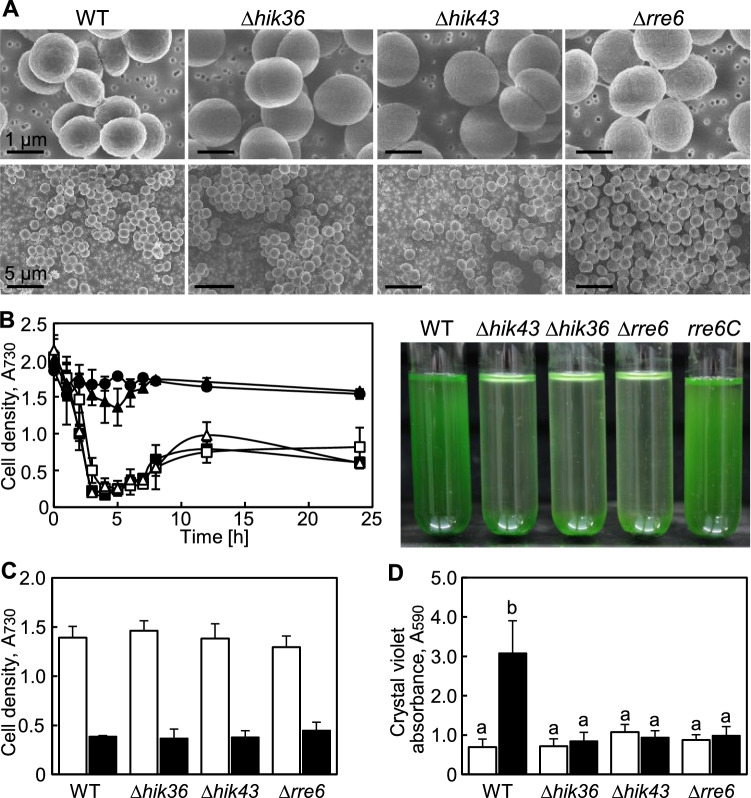
Phenotypes of *Synechocystis* mutants. (**A**) Representative FE-SEM images of wild type (WT), Δ*hik36*, Δ*hik43* and Δ*rre6 Synechocystis* cells. (**B**) Cell sedimentation of WT (filled circles), Δ*hik36* (filled squares), Δ*hik43* (open squares), Δ*rre6* (open triangles) and *rre6C* (filled triangles). Samples were taken at the surface of cultures left without shaking and OD_730_ was determined. Each value corresponds to mean ± SD (n = 3) (left). Representative images of cell cultures after being left to settle for 24 h (right). (**C**) Growth of WT, Δ*hik36*, Δ*hik43*, Δ*rre6*, and *rre6C* in media without (white bars) and with (black bars) 500 mM NaCl for 3 days. Each value corresponds to mean ± SD (n = 5). (**D**) Biofilm formation of WT, Δ*hik36*, Δ*hik43* and Δ*rre6* in media without (white bars) and with (black bars) 500 mM NaCl for 3 days. Each value corresponds to mean ± SD (n = 8–13). Significant differences between WT and each mutant were analyzed by Tukey's test (* *p* < 0.05).

Hik36–Hik43 and Rre7 are known to form at TCS^28^, but a TCS pair consisting of Hik36–Hik43 and Rre6 has not been studied to date (Fig. 1). To evaluate a potential physiological connection of Hik36–Hik43 and Rre6, individual mutants (*Δhik36*, *Δhik43* and *Δrre6*) were generated in *Synechocystis* by insertion of a kanamycin or a spectinomycin resistance gene cassette into the genome sequence (Fig. 2). Reintroduction of the coding sequence of *hik36* and *hik43* into the native locus in *Δhik36* or *Δhik43*, respectively, failed, presumably due to the low transformation efficiency of the mutants^28^. In contrast, we were able to reintroduce *rre6* into *Δrre6,* which reflected the normal transformation competency of *Δrre6*^28^. The complemented strain was designated *rre6C*. The morphology of wild-type and mutant cells was observed by field emission-scanning electron microscopy (FE-SEM). All mutants were similar to the wild type with respect to size and cell shape (Fig. 3A). We noticed that the disruption mutants spontaneously aggregated and sedimented at the bottom of test tubes when they were left without agitation in the light, a process termed autoaggregation^1^. Therefore, the sedimentation rates of *Δhik36*, *Δhik43*, *Δrre6* and *rre6C* and the wild type were evaluated in static cultures (Fig. 3B). Sedimentation occurred rapidly in *Δhik36*, *Δhik43* and *Δrre6* cultures, which appeared transparent after 4 h (OD_730_ = less than 0.5). In contrast, the wild type and the complemented strain *rre6C* did not sediment even after 24 h (OD_730_ = more than 1.5 at 24 h). These results suggested that presence of Rre6 and therefore of a TCS consisting of Hik36–Hik43–Rre6 prevented cell aggregation in the wild-type and *rre6C* cultures.

Since *Synechocystis* forms biofilms under salt stress conditions^20^, *Δhik36*, *Δhik43*, *Δrre6* and the wild type were cultured without or with added NaCl (500 mM). After growth for 30 days, the cell density of *Δhik36*, *Δhik43* and *Δrre6* cultures was similar to that of the wild type in both conditions (Fig. 3C). Biofilm formation in the wild type increased during salt stress, but no such increase was seen in *Δhik36*, *Δhik43* and *Δrre6* (Fig. 3D). Cells of *Δhik36*, *Δhik43* and *Δrre6* aggregated but were unable to attach to solid materials like the glass or plastic walls of the culture vessels. These results indicated that Hik36–Hik43–Rre6 promoted biofilm production during salt stress.

### Expression of EPS-producing genes was changed in *Δhik43*, *Δhik36* and *Δrre6*

**Figure 4:**
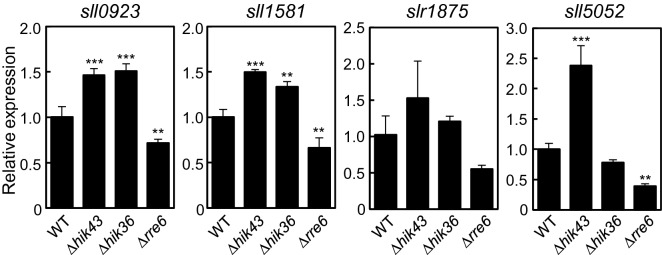
Expression of EPS production genes. mRNA levels of *sll0923*, *sll1581*, *slr1875* and *sll05052* were determined by quantitative RT-PCR analysis using the 2^−ΔΔCT^ quantification method and *rnpB* as internal control. Expression levels are shown as relative values to those of the WT. Each value corresponds to mean ± SD (n = 3). Significant differences between WT and mutants were analyzed by Dunnett’s test (** *p* < 0.01, *** *p* < 0.001).

EPS help to protect cells against salt stress and some mutants affected in EPS production display increased cell-to-cell aggregation^19^. We therefore evaluated whether expression of the four EPS production genes, *sll0923*, *sll1581*, *slr1875*, and *sll5052* was changed in *Δhik43*, *Δhik36* and *Δrre6*^19^ (Fig. 4). Expression of all four genes increased in *Δhik43.* In *Δhik36* expression of *sll5052* was the same as in the wild type, but expression of the other three genes also increased. The differences in accumulation of *sll5052* transcripts in *Δhik36* and *Δhik43* may be caused by a difference in the function of Hik36 and Hik43. In contrast, expression of all four genes decreased in *Δrre6*. These results suggest that loss of *hik36*, *hik43* and *rre6* affects expression of the EPS production genes (Fig. 3B).

### Rre6 interacted with PilT2

**Figure 5:**
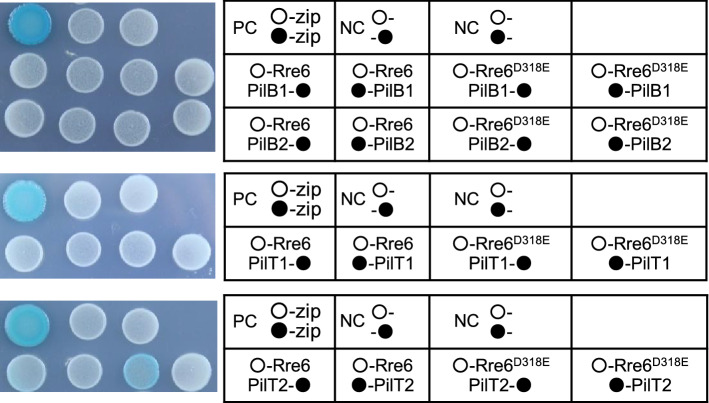
Analysis of protein–protein interactions of Rre6 with PilB1/2 and PilT1/2. Bacterial two-hybrid assays. Rre6 and Rre6^D318E^ were expressed as T25-fusion proteins. PilB1, PilB2, PilT1 and PilT2 were expressed as T18-fusion proteins. Colonies were grown on solid medium containing 0.5 mM IPTG and 80 mg/mL X-Gal for 48 h. T25 fragments expressed by pKT were shown as ○-. T18 fragments expressed by pUT18C or pUT18 were shown as ●- or -●, respectively. *Escherichia coli* containing pKT25-zip and pUT18C-zip, or pKT25-zip and pUT18-zip were used as positive control (PC), and the cells containing pKT25 and pUT18, or pKT25 and pUT18C were used as negative controls (NC).

The motility of *Synechocystis* is controlled by pili, whose biogenesis, assembly, retraction and function requires dozens of genes^31,32^. Based on the gene assignment of the type IV pili (TFP) biogenesis and signal transduction system in *Synechocystis*, we hypothesized that Rre6 might interact with two ATPases, PilB and PilT, that are membrane peripheral proteins of the pilus system, localized on the cytoplasmic side of the inner membrane. *Synechocystis* contains two copies of both PilB (PilB1, PilB2) and PilT (PilT1, PilT2)^12,13^. A BACTH assay was performed to analyze possible protein–protein interactions (Fig. 5). Of all combinations tested (Rre6–PilB1, Rre6–PilB2, Rre6–PilT1 and Rre6–PilT2), only Rre6–PilT2 resulted in light blue colonies, indicating that these two proteins interacted with each other (Fig. 5). To examine whether the interaction between Rre6 and PilT2 was dependent on phosphorylation of Rre6, we converted the predicted phosphorylated residue D318 in Rre6 to E (phosphomimic: Rre6^D318E^). *E. coli* co-expressing Rre6^D318E^/PilT2 resulted in blue colonies but colonies expressing Rre6/PilT2 remained white (Fig. 5). These data suggest that Hik36–Hik43-mediated phosphorylation of Rre6 enabled interaction of Rre6 with PilT2.

## Discussion

**Figure 6:**
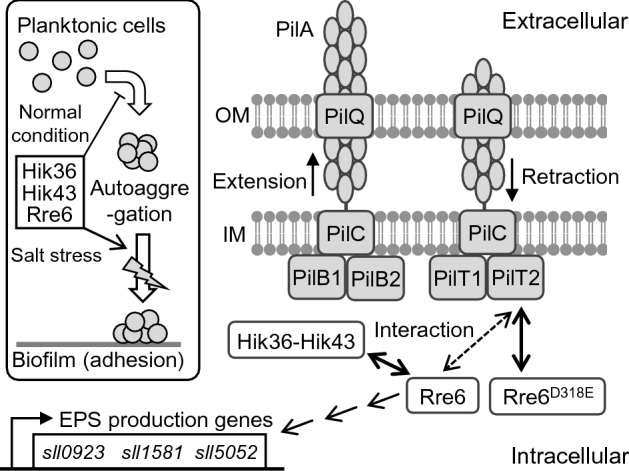
Model for Hik36–Hik43–Rre6 functions in TFP regulation and biofilm formation in *Synechocystis*. Hik36–Hik43–Rre6 prevents autoaggregation but promotes biofilm formation under high salinity conditions. According to the model by Bertrand et al.^9^ and Schuergers and Wilde^32^, PilB and PilT conduct the regulation of pilus extension and retraction, respectively. The major pilin PilA forms the filament and PilB1 assembles the filament. PilT depolymerizes TFP. Hik36–Hik43 phosphorylates Rre6. The phosphomimic Rre6^D318E^ interacts with PilT2. OM, outer membrane; IM, inner membrane. Rre6 affects the expression of EPS production genes, *sll0923*, *sll1581* and *sll05052*^19^.

*Synechocystis* is known to induce biofilm formation as an adaptation to environmental changes^19,20^. The signal transduction pathway regulating this process remains to be elucidated. This study investigated the role of members of the *tax3*/*pilG* cluster, namely two genes encoding response regulators, *rre6* and *rre7*, as well as two genes, *hik36* and *hik43*, that together encode a histidine kinase. We showed that Hik36–Hik43 and Rre6 comprise a TCS, and function in the prevention of autoaggregation in *Synechocystis* (Fig. 6). Yoshihara et al. predicted that Rre7 was the receiver for Hik36–Hik43 in the phosphotransfer reaction due to the fact that the *tax3*/*pilG* cluster has a similar genome arrangement to the corresponding cluster in *P. aeruginosa*^28^. Contrary to this prediction, our study showed that Hik36–Hik43 physically interacted with Rre6, but not with Rre7. However, we cannot exclude the possibility that Rre7 might also interact with Hik36–Hik43. Rre6 (388 amino acids) and Rre7 (146 amino acids) share high homology in their receiver domains, but Rre6 has an extended N-terminal sequence which is absent from Rre7^28^. This difference of the N-terminal regions might be responsible for the difference in function between Rre6 and Rre7. Hik36 contains a histidine phosphotransfer domains (HPt), responsible for phosphotransfer to an aspartate on a response regulator^28^. The interaction between Hik36 and Rre6 in the BACTH assay (Fig. 1) therefore suggested a possible phosphotransfer reaction between them. In contrast, the physiological function of the detected interaction between Hik43 and Rre6 remains to be elucidated.

According to the proposed model of the Chp-chemosensory system in *Pseudomonas aeruginosa*^9^, PilB and PilT are peripheral membrane proteins of the TFP system that function as motor proteins with ATPase activity (Fig. 6). PilB drives pilus extension, and PilT conducts pilus retraction. Our study detected interaction between Rre6 and PilT2, but not PilB. A phosphomimic form, Rre6D^318E^ was able to bind to PilT2, indicating that Rre6 is part of the signal transduction pathway regulating TFP function (Fig. 5).

Cell aggregation and sedimentation enhance biofilm formation, and the process of cells shifting from a planktonic state to a biofilm involves multiple steps^1^. In *Synechococcus elongatus* PCC 7942, loss of pili promotes cell sedimentation and biofilm development^33^. In *Synechocystis*, absence of the major pilin PilA1 has little effect on flocculation and TFP are not absolutely required for biofilm development^34^. *Δhik36*, *Δhik43* and *Δrre6* showed an increased rate of sedimentation compared with the wild type, but unlike the wild type they did not form biofilms under salt stress conditions (Fig. 3). This inconsistency between cell aggregation and biofilm formation has been observed in other bacteria^35^. The outer membrane-associated ligand binding protein, BipA in *Bordetella holmesii* prevents autoaggregation but promotes biofilm production^35^. In *Synechocystis*, *sll1581* deletion mutants show increased autoaggregation but less adhesion to glass surfaces because of a reduction of electrostatic repulsion around the cells^36^. *Synechocystis* has pili, S-layer and EPS as cell surface structures, which all affect cell to cell interactions and adhesion to solid surfaces in the culture. The rate of sedimentation of *Synechocystis* is regulated by electrostatic repulsion, which depends on net charges of the EPS^36^. The expression of EPS genes in *Δhik36*, *Δhik43* and *Δrre6* differed from those of the WT (Fig. 4). Therefore, loss of function of the TCS might lead to a disruption of the normal cell surface net charge. Lack of Sll1581 impairs biofilm formation, and loss of Sll0923 decreases EPS by approximately 50%^36^. Our data indicate that Rre6 promoted the expression of the EPS production genes, *sll0923*, *sll1581* and *sll5052*^19^ (Fig. 4). Decrease of the amount of EPS in *Δrre6* may account for a loss of repulsion, resulting in autoaggregation. In the case of *Δhik36* and *Δhik43*, production of different amounts of individual EPS components might also result in different net charges of the cell surface.

A domain search in Rre6 using SMART (https://smart.embl-heidelberg.de/) and Pfam (https://pfam.xfam.org/) found that Rre6 has only one REC domain without DNA binding motif and enzymatic domain. This is similar to the response regulator PilG from *Pseudomonas aeruginosa*, a homolog of Rre6, which also contains only one REC domain. PilG pairs with the histidine kinase ChpA in a TCS that stimulates the membrane bound-adenylated cyclase, CyaB^37,38^. *Synechocystis* has two adenylate cyclase genes, *cya1* and *cya2*. Mutational analysis revealed that *cya1*is essential for cell motility, and application of cyclic AMP to the cells confirmed that cyclic AMP is involved in regulation of cell motility^39^. Understanding the connection of Rre6 with cyclic AMP may be a clue to understanding the signaling pathway regulating the expression of the EPS production genes *sll0923*, *sll1581* and *sll5052*.

The pili of *Synechocystis* can be classified into two types, thick pili and thin pili^12^. The pilus structure of *Δrre6* (*ΔpilG*) is the same as that of the wild type. *Δhik36* (*ΔpilL-N*) has longer and more numerous thick pili, and *Δhik43* (*ΔpilL-C*) has almost no thick pili, but both mutants possess normal thin pili^13,28^. The data indicated that these mutations affect pilus structure differently although Hik36, Hik43 and Rre6 are part of the same TCS (Fig. 6). A comprehensive study of *E. coli* TCS revealed that one Hik can form a pair with multiple Rre and that one Rre can form a pair with multiple Hik^40^. It is therefore possible that other Hik or Rre could also participate in the phospho-relay of Hik36, Hik43 and Rre6, at least with respect to pilus formation. Deletion of PilT1 resulted in a drastic increase in the number of thick pili, whereas deletion of PilT2 had no effect on the number and shape of pili^12,13^. Deletion of either Rre6 or PilT2 resulted in the same phenotype and neither was involved in regulation of pilus structure. This was also consistent with the fact that Rre6^D318E^ interacted with PilT2 (Fig. 5).

Our study indicates that Hik36–Hik43–Rre6 form a TCS, and that the phosphomimic form, Rre6D^318E^ can physically interact with PilT2. Hik36–Hik43–Rre6 function as an upstream component of the pili-related signal transduction cascade and control the prevention of cell adhesion and biofilm formation. Further studies will be required to work out the details of in vivo protein–protein interactions between Rre6 and Hik36–Hik43 and to fully understand the molecular switch involved in cell sedimentation and cell motility in response to complex environmental changes, for example changes in light and salinity.

### Experimental procedures

#### Cells and growth conditions

*Synechocystis* sp. PCC 6803 GT-strain was grown at 29 °C in BG11 medium with shaking and continuous light (50 µE m^−2^ s^−1^)^41^. Growth was monitored by measuring the OD_730_ of the culture.

#### Crystal violet assay

Crystal violet assay was performed as previously described^20^. Briefly, cells were cultured in liquid BG11 medium with or without NaCl in a 96-well polystyrene microtiter plate (Merck, https://www.merckgroup.com/en) at 29 °C. The cells attached to the microtiter plate wells were stained with crystal violet. The bound crystal violet was then extracted with 70% ethanol and the absorbance was measured at 590 nm using a plate reader.

#### Detection by field emission scanning electron microscopy (FE-SEM)

Cells were gently suspended in PBS and fixed in 1.25% glutaraldehyde for 12 h at 4 °C. After dehydration through an ethanol series (from 0 to 100%) and *t*-butyl alcohol, cells were transferred to polycarbonate membranes and lyophilized in a vacuum evaporator. The membranes were sputter-coated with osmium (POC-3 osmium plasma coater, MEIWAFOSIS Co., https://www.meiwafosis.com/) and examined under a FE-SEM (S-4800; Hitachi, https://www.hitachi.com/).

### Site-directed mutagenesis of Rre6

The 318th codon (GAC) of *rre6*, encoding aspartate (D318) was changed to GAA, encoding glutamate (D318E) as a phosphomimic or to GCC, encoding alanine (D318A) as a mimic of the dephosphorylated form using PCR. To generate *rre6*^D318E^, two fragments were generated using primers rre6_XbaI_Fw and rre6_D318E_Rv, or rre6_D318E_Fw and rre6_XbaI_Rv and then fused by PCR using primers rre6_XbaI_Fw and rre6_XbaI_Rv. To generate *rre6*^D318A^,two fragments were generated using rre6_XbaI_Fw and rre6_D318A_Rv, or rre6_D318A_Fw and rre6_XbaI_Rv and then fused by PCR using rre6_XbaI_Fw and rre6_XbaI_Rv. Each *rre6* variant was subcloned into the *Xba*I site of pUC19 and the mutations were confirmed by DNA sequencing. All primer sequences are listed in Supplemental Table S1.

### Inactivation and reintroduction of genes in *Synechocystis* sp. PCC 6803

A kanamycin resistance gene (*Km*^*r*^) amplified with pUC19_Km^r^_Fw and pUC19_Km^r^_Rv or a spectinomycin resistance (*Spm*^*r*^) gene amplified with pUC19_Spm^r^_Fw and pUC19_Spm^r^_Rv was cloned into pUC19, respectively. The resultant plasmids were designated pUC19_Spm^r^ and pUC19_Spm^r^. To disrupt *hik36* (Δ*hik36*), *hik43* (Δ*hik43*) or *rre6* (Δ*rre6*) in *Synechocystis*, the coding sequence from start to stop codon of each individual gene was replaced by the *Km*^*r*^ or *Spm*^*r*^ gene cassette in this way: The upstream region of *hik36,* amplified using hik36_U_Fw and hik36_U_Rv, and the downstream region of *hik36,* amplified using hik36_D_Fw and hik36_D_Rv, were inserted into *Eco*RV site or *Hpa*I site of pUC19_Spm^r^_._ The upstream region of *hik43* amplified using hik43_U_Fw and hik43_U_Rv, and the downstream region of *hik43* amplified using, hik43_D_Fw and hik43_D_Rv were inserted into *Eco*RV site or *Hpa*I site of pUC19_Spm^r^_._ The upstream region of *rre6* amplified using rre6_U_Fw and rre6_U_Rv, and the downstream region of *rre6* amplified using rre6_D_Fw and rre6_D_Rv were inserted into *Eco*RV site or *Nco*I site of pUC19_Km^r^_._ These plasmids were introduced into *Synechocystis* to disrupt *hik36* (Δ*hik36*), *hik43* (Δ*hik43*) or *rre6* (Δ*rre6*). After segregation in BG11 medium containing kanamycin (25 µg/mL) or spectinomycin (20 µg/mL), the gene disruptions were confirmed by PCR using hik36_U_Fw and hik36_D_Rv, hik43_U_Fw and hik43_D_Rv, or rre6_U_Fw and rre6_D_Rv with genomic DNA as template.

For reintroduction of *rre6* into the native locus, the upstream and the coding sequence of *rre6* amplified using rre6_U_Fw and rre6_U_Rv2, was introduced into *Eco*RV site of pUC19_Spm^r^. The downstream sequences of *rre6* amplified using rre6_D_Fw2 and rre6_D_Rv2 was introduced into *Hpa*I site of the same plasmid. Reintroduction of *rre6* with *Spm*^*r*^ gene was performed by integration into the *Km*^*r*^ gene in *Δrre6*. Upon segregation in BG11 medium containing spectinomycin (20 µg/mL), the correct integration was confirmed by PCR using rre6_U_Fw and rre6_D_Rv. The resultant strains were named *rre6C*. The primer sequences used in this experiment are listed in Supplemental Table S1.

### Bacterial two-hybrid assay

A bacterial two-hybrid assay was performed according to the Euromedex manual (Euromedex, https://web.euromedex.com/). For the analysis of interactions among TCSs, *hik36* was amplified using hik36_pKNT25_Fw and hik36_pKNT25_Rv and introduced into the *Bam*HI-*Kpn*I sites of pKNT25. The *hik36* fragment, digested by *Bam*HI and *Kpn*I was also introduced into the *Bam*HI-*Kpn*I sites of pUT18C. The *hik43*, *rre6* and *rre7* were amplified using a pair of primers listed in Supplemental Table S1 and introduced into the *Bam*HI-*Kpn*I sites of pUT18C. The *hik43* fragment and *rre6* fragment digested by *Bam*HI and *Kpn*I was further introduced into *Bam*HI-*Kpn*I site of pKNT25. The 614 amino acids of C-terminal deletion mutant of *hik43* (*hik43*Δ*C*) was amplified using hik43_pUT18C_Fw and hik43ΔC_pUT18C_Rv, and the 360 amino acids of N-terminal deletion mutant of *hik43* (*hik43*Δ*N*) was amplified using hik43ΔN_pUT18C_Fw and hik43_pUT18C_Rv, and introduced into *Bam*HI-*Kpn*I site of pUT18C. The primer sequences used in this experiment are listed in Supplemental Table S1. For the analysis of interactions between Rre6 and pilin related proteins pilB1, pilB2, pilT1, and pilT2, *rre6* and *rre6*^D318E^ were amplified using rre6_pKT25_Fw and rre6_pKT25_Rv, and introduced into *Sma*I site of pKT25, respectively. The *pilB1*, *pilB2*, *pilT1*, *and pilT2* sequences were amplified using a pair of corresponding primers listed in Table S1 and the PCR products were introduced into the *Sal*I site of pUT18 or pUT18C, respectively. *Escherichia coli* BTH101 cells were co-transformed with each pK and pUT construct. The precultured transformed cells were diluted to an OD_600_ = 0.005 and 5 µL of them were spotted onto solid Luria–Bertani (LB) medium containing 0.5 mM IPTG and 80 mg/mL X-Gal.

### Quantitative RT-PCR

Total RNA was extracted with TRI Reagent (Merck, https://www.merckgroup.com/en) following the manufacturer’s protocol. cDNA was synthesized using ReverTra Ace quantitative PCR reverse transcription master mix with genomic DNA Remover (Toyobo, https://www.toyobo-global.com/). Gene expression levels were analyzed by quantitative RT-PCR using the 2^−ΔΔCT^ quantification method and *rnpB* as internal control. The primer sequences used in this experiment are listed in Table S1.

### Measurement of autoaggregation

Cells were cultured in BG11 medium until they reached OD_730_ = 0.5–1.0, and then adjusted to OD_730_ = 3.0 in 10 mL of BG11 medium in test tubes. Cells were left in the growth chamber at 29 °C in continuous light. At every time point, 50 µl of culture were taken from the surface of the liquid.

## Supplementary information

Supplementary Information

## Abbreviations

BACTH: Bacterial two-hybrid
CA: Catalytic and ATP binding
CWI: CheW interaction
DIM: Homodimeric
EPS: Exopolysaccharides
FE-SEM: Field emission-scanning electron microscopy
Hik: Histidine kinase
HPT: Histidine phosphotransfer
REC: Receiver
RT-PCR: Real time-polymerase chain reaction
TCS: Two-component regulatory system
TFP: Type IV pilus
