# Supplementary material for: Hik36–Hik43 and Rre6 act as a two-component regulatory system to control cell aggregation in Synechocystis sp. PCC6803
Source: Sci Rep. 2020 Nov 10;10:19405. doi: 10.1038/s41598-020-76264-2 (PMC7656254; doi:10.1038/s41598-020-76264-2)
Supplement: Supplementary file 1 — Supplementary Information [file 41598_2020_76264_MOESM1_ESM.pdf]

Hik36-Hik43 and Rre6 act as a two-component regulatory system to control cell aggregation in *Synechocystis* sp. PCC6803

Kota Kera, Yuichiro Yoshizawa, Takehiro Shigehara, Tatsuya Nagayama, Masaru Tochigi, Saeko Tochigi and Nobuyuki Uozumi§

Department of Biomolecular Engineering, Graduate School of Engineering, Tohoku University, Sendai, Japan

## Supplemental Table S1. Primers used in this study

| | Gene name | Primer name | Sequence (5' - 3') |
|---|---|---|---|
| Site-directed mutagenesis | *rre6* (*slr1041*) | rre6_XbaI_Fw | GCAGGTCGACTCTAGTAATGCGGTCGGAGTTGCAT |
| | | rre6_D318E_Fw | CTTTGTGAAATCACCATGCCCCACCTG |
| | | rre6_D318E_Rv | GGTGATTTCACAAAGGATCAGATCAGGCTGC |
| | | rre6_D318A_Fw | CTTTGTGCCATCACCATGCCCCACCTG |
| | | rre6_D318A_Rv | GGTGATGGCACAAAGGATCAGATCAGGCTGC |
| | | rre6_XbaI_Rv | TCGGGGATCCTCTAGTCATGATTTTTTACAGTAAGATTCAACTAA |
| Disruption of genes | *Km*ʳ | pUC19_Kmʳ_Fw | CTCTAGAGGATCCCCGATATCGAATTCAGATCTGTTAACAGCTGA |
| | | pUC19_Kmʳ_Rv | TCGAGCTCGGTACCCCCATGGGAATTCAGATCTGTTAACAGCTGA |
| | *Sp*ʳ | pUC19_Spʳ_Fw | CTCTAGAGGATCCCCGATATCGTCGACTCTCCCAATTTGTGTAG |
| | | pUC19_Spʳ_Rv | TCGAGCTCGGTACCCGTTAACGTCGACGGATCAATTCCCCTG |
| | Upstream of *hik36* | hik36_U_Fw | CAGAGGATCCCCGATGGTTGTCTGTGCGGAATATGC |
| | | hik36_U_Rv | CTTTAGCTCGCTGGCGGTATATCGTCGACTCTCCC |
| | Downstream of *hik36* | hik36_D_Fw | TGATCCGTCGACGTTTTCGGGGTTTTAGACTGGATCAAA |
| | | hik36_D_Rv | AGCTCGGTACCCGTTTCAACAGGAAAGACAGAGCATGG |
| | Upstream of *hik36* | Hik43_U_Fw | CAGAGGATCCCCGATGAAGAACTTAACGCTAGCTATGAAACC |
| | | Hik43_U_Rv | GTGTGGAAGAGTAGCAAGAAGCAATCGTCGACTCTCCC |
| | Downstream of *hik43* | Hik43_D_Fw | TGATCCGTCGACGTTCAGAATCGTTGAGTCCCTTACTGC |
| | | Hik43_D_Rv | AGCTCGGTACCCGTTGAACTTCAATTTCGTCAGCAATAAAACC |
| | Upstream of *rre6* | rre6_U_Fw | TAGAGGATCCCCGATCGAGCCGATGACCAGATTAAGG |
| | | rre6_U_Rv | TACACATCAATCTTGTGAAAACGCCATCGAATTCAGATCT |
| | Downstream of *rre6* | rre6_D_Fw | ATCTGAATTCCCATGTCTTTTGATCATTATCTCCATCCCCC |
| | | rre6_D_Rv | CCTACATTGCCAAACCATTTCAGCCATGGGGGTACCGAG |
| Reintroduction of genes | Upstream and *rre6* | rre6_U_Fw | TAGAGGATCCCCGATCGAGCCGATGACCAGATTAAGG |
| | | rre6_U_Rv2 | GGGAGAGTCGACGATTCATGATTTTTTACAGTAAGATTCAACTAA |
| | Downstream of *rre6* | rre6_D_Fw2 | TGATCCGTCGACGTTTCTTTTGATCATTATCTCCATCCCC |
| | | rre6_D_Rv2 | AGCTCGGTACCCGTTCACCAGTTCCATGGGCTGAAA |
| Genotyping | *hik36* (slr0073) | hik36_U_Fw | CAGAGGATCCCCGATGGTTGTCTGTGCGGAATATGC |
| | | hik36_D_Rv | AGCTCGGTACCCGTTTCAACAGGAAAGACAGAGCATGG |
| | *hik43* (*slr0322*) | hik43_U_Fw | CAGAGGATCCCCGATGAAGAACTTAACGCTAGCTATGAAACC |
| | | hik43_D_Rv | AGCTCGGTACCCGTTGAACTTCAATTTCGTCAGCAATAAAACC |
| | *rre6* (*slr1041*) | rre6_U_Fw | TAGAGGATCCCCGATCGAGCCGATGACCAGATTAAGG |
| | | rre6_D_Rv | CCTACATTGCCAAACCATTTCAGCCATGGGGGTACCGAG |

| | | | |
|---|---|---|---|
| Bacterial two-hybrid assay | *hik36* (*slr0073*) | hik36_pKNT25_Fw | AAAAAAGGATCCCCTAGGGGAGTGGCCATCCAAAT |
| | | hik36_pKNT25_Rv | AAAAAAGGTACCCGACGGGCGGCAGAAAGATTTTTAAT |
| | *hik43* (*slr0322*) | hik43_pUT18C_Fw | GCAGGTCGACTCTAGAGACTAGCGATCCCAATCCAGC |
| | | hik43_pUT18C_Rv | CCGGGGATCCTCTAGCTCGTCTGCACTTAGAGCGG |
| | | hik43DN_pUT18C_Fw | GCAGGTCGACTCTAGAGAATAAAAAAGTCAACCTAGAGGTGGAAG |
| | | hik43DC_pUT18C_Rv | CCGGGGATCCTCTAGCTCCAAAATCATTTTGTCGATGAGCAC |
| | *rre6* (*slr1041*) | rre6_pUT18C_Fw | AAAAAATCTAGAGCAGGGAACCCTGAACGAAATT |
| | | rre6_pUT18C_Rv | TTTTTTGGTACCCGTGATTTTTTACAGTAAGATTCAACTAATG |
| | | rre6_pKT25_Fw | CTCTAGAGGATCCCCGGATGCAGGGAACCCTGAAC |
| | | rre6_pKT25_Rv | GTTACTTAGGTACCCTCATGATTTTTTACAGTAAGATTCAACTAA |
| | *rre7* (*slr1042*) | rre7_pUT18C_Fw | TTTTTTGGATCCCAACGCAGTTTTGCTGGTTGAAG |
| | | rre7_pUT18C_Rv | AAAAAAGGTACCCGATTGCGCAGGAGTTGTTTGATG |
| | *pilB1* (*slr0063*) | pilB1_pUT18_Fw | GCTTGCATGCCTGCAGGTCGACTATGACATCTTCCTCCTCTTCC |
| | | pilB1_pUT18_Rv | CGGGGATCCTCTAGAGTCGACCTGCTAAACCGGGAAGTCATGC |
| | | pilB1_pUT18C_Fw | GGAACGCCACTGCAGGTCGACTATGACATCTTCCTCCTCTTCC |
| | *pilB2* (*slr0079*) | pilB2_pUT18_Fw | GCTTGCATGCCTGCAGGTCGACTATGGTCTTTTCCTCCGATTCC |
| | | pilB2_pUT18_Rv | CGGGGATCCTCTAGAGTCGACCTCCAGGGGTCAATCACAGATTTA |
| | | pilB2_pUT18C_Fw | GGAACGCCACTGCAGGTCGACTATGGTCTTTTCCTCCGATTCC |
| | *pilT1* (*slr0161*) | pilT1_pUT18_Fw | GCTTGCATGCCTGCAGGTCGACTATGGCTTTGGAATACATGATCG |
| | | pilT1_pUT18_Rv | CGGGGATCCTCTAGAGTCGACCTACGACGTTTAGCGGCAAC |
| | | pilT1_pUT18C_Fw | GGAACGCCACTGCAGGTCGACTATGGCTTTGGAATACATGATCG |
| | *pilT2* (*sll1533*) | pilT2_pUT18_Fw | GCTTGCATGCCTGCAGGTCGACTATGAACCAACCTCCCCGC |
| | | pilT2_pUT18_Rv | CGGGGATCCTCTAGAGTCGACCTGGTTCTGCCCCGCAGTC |
| | | pilT2_pUT18C_Fw | GGAACGCCACTGCAGGTCGACTATGAACCAACCTCCCCGC |
| RT-PCR | *sll0923* | sll0923_RT_Fw | TTGGCGTTGGCTGATCTCAA |
| | | sll0923_RT_Rv | CATAACCGCCGGACGTAGAA |
| | *sll1581* | sll1581_RT_Fw | ACGGCGACGTCATTACAGTT |
| | | sll1581_RT_Rv | CTGGATAGCACCACCTCGTC |
| | *slr1875* | slr1875_RT_Fw | TCTCCAGGAGGATGATGGCT |
| | | slr1875_RT_Fw | TGATGGTAATACCGCCGTTGA |
| | *sll5052* | sll5052_RT_Fw | AGCCGAATTACCATGCTGGA |
| | | sll5052_RT_Rv | CGTATTGAGCCGCAATGGTG |
| | *rnpB* | rnpB_RT_Fw | AGGGTAAGGGTGCAAAGGTG |
| | | rnpB_RT_Rv | GTCGTAAGCCGGGTTC |

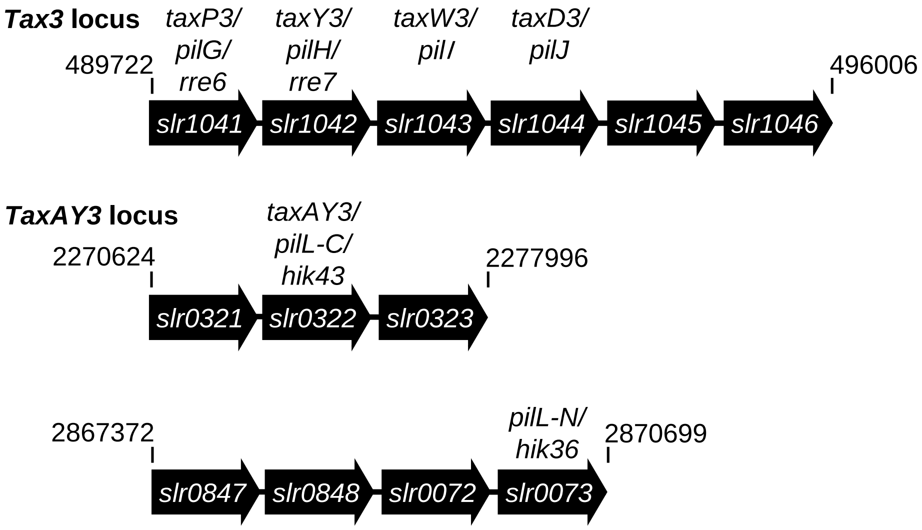


**Supplemental Fig.S1 Gene organization of *hik36*, *hik43 rre6* and *rre7*.** The numbers indicate the position in the *Synechocystis* genome. The schematic model was derived from the model by Bhaya et al [27] and Yoshihara et al [28].
